# Synthesis, Spectral, Thermal and Biological Studies of 4-Cyclohexyl-3-(4-nitrophenyl)methyl-1,2,4-triazolin-5-thione and Its Copper(II) Coordination Compound, [CuCl_2_(H_2_O)_2_L_2_]

**DOI:** 10.3390/ma13184135

**Published:** 2020-09-17

**Authors:** Agnieszka Czylkowska, Monika Drozd, Anna Biernasiuk, Bartłomiej Rogalewicz, Anna Malm, Monika Pitucha

**Affiliations:** 1 Institute of General and Ecological Chemistry, Faculty of Chemistry, Lodz University of Technology, Zeromskiego 116, 90-924 Lodz, Poland; 211150@edu.p.lodz.pl; 2 Independent Radiopharmacy Unit, Faculty of Pharmacy, Medical University of Lublin, Chodzki 4A, 20-093 Lublin, Poland; monika.drozd@umlub.pl (M.D.); monika.pitucha@umlub.pl (M.P.); 3 Department of Pharmaceutical Microbiology with the Laboratory of Microbiological Diagnostics, Faculty of Pharmacy, Medical University of Lublin, Chodzki 4A, 20-093 Lublin, Poland; anna.biernasiuk@umlub.pl (A.B.); anna.malm@umlub.pl (A.M.)

**Keywords:** copper(II) complex, triazole derivative, antimicrobial activity, FTIR, TG-DTG

## Abstract

One of the strategies for seeking new biologically active substances is to modify compounds with potential biological activity. In this paper, 1,2,4-triazolin-5-thione derivative (**3**) was obtained in the cyclization reaction of appropriate thiosemicarbazide (**2**) as an organic ligand. The copper(II) complex, [CuCl_2_(H_2_O)_2_L_2_] (L=4-cyclohexyl-3-(nitrophenyl)methyl-1,2,4-triazolin-5-thione) (**Cu-3**) was prepared in a reaction of free ligand (**3**) with a CuCl_2_·2H_2_O solution in MeOH/EtOH mixture at room temperature. TGA data show that **Cu-3** and free ligand are stable at room temperature. Both compounds were screened in vitro for antibacterial and antifungal activities using the broth microdilution method. The obtained complex (**Cu-3**) showed higher antibacterial effect, especially towards Gram-positive bacteria (with moderate activity and Minimal Inhibitory Concentration MIC = 250–500 µg/mL) than the free ligand (**3**) (with mild or no bioactivity and MIC ≥ 1000 µg/mL). In turn, yeasts, belonging to *Candida albicans*, exhibited similar sensitivity to both the copper(II) complex (**Cu-3**) and the organic ligand (**3**). The anticandidal activity of these compounds was moderate (MIC = 500 µg/mL), or, in the case of other *Candida* spp., lower (MIC ≥ 1000 µg/mL).

## 1. Introduction

Drugs are chemical compounds that are used to obtain a certain reaction in the functioning of an organism through their biological or chemical properties. Medicines in different forms have been used for years and still represent an area of great interest and opportunities. Drugs can sometimes have a simultaneous negative effect on a human organism. Some of the most common drawbacks of using drugs are unwanted side effects, low efficiency and possible drug addiction; however, this problem can be solved through discovering new medicines or modifying drugs that are already in use [1,2]. Coordination chemistry creates a great opportunity to obtain new coordination compounds that may display better properties than drugs used in their standard form [3,4,5,6,7,8,9,10,11,12]. To reach this goal, drugs are used as ligands to form complex derivatives. In such derivatives, centers of coordination are usually biologically important metal ions [13,14]. New coordination compounds are tested for their biological, antibacterial, antiviral or antifungal properties [4,15,16,17,18]. Drug complexes may also be used as radiopharmaceuticals or fluorescence sensors [19,20]. Such an approach to this problem allows researchers to obtain and investigate a huge amount of different combinations of complex centers and ligands, hence increasing the probability of receiving outstanding results. 

Copper(II) complexes with heterocyclic ligands with proven or potential biological activity appear to be an attractive group of compounds [21,22,23,24,25]. In this context, triazole derivatives seem to be interesting, as they have a wide spectrum of biological activity and have long been used as medicines. For example, synthetic triazoles, antifungal agents; fluconazole, itraconazole, and 2nd generation of triazoles; voriconazole, posaconazole or rawuconazole. 

In our work, we choose copper because, according to many authors, copper has various biological properties, especially antibacterial, antifungal and antiviral. Therefore, various forms of copper (ions, either alone or in complexes), are used for the disinfection of liquids, solids and human tissues. This metal is also used as a water purifier, fungicide, algaecide, molluscicide, nematocide, as well as an antifouling agent. The antimicrobial properties of copper depend on its form—solid, ionic or particles [26,27,28]. Copper can be used in solid form in different medical environments—for example, in hospitals on various inanimate surfaces, in different facilities, or for medical devices. To test its antimicrobial surface activity, researchers mostly use coupons with varying concentrations of metal. In such studies, the high antibacterial activity of copper coupons towards clinical isolates of *Escherichia coli*, *Klebsiella pneumoniae*, *Enterobacter* spp., *Acinetobacter baumannii* and *Pseudomonas aeruginosa*–potent multidrug-resistant Gram-negative pathogens responsible for nosocomial infections was investigated. The use of copper in the form of nanoparticles—for example chitosan-copper nanoparticles also showed great antibacterial effectiveness on many strains including *Bacillus subtilis*, *Staphylococcus aureus*, *Salmonella choleraesuis* and *P. aeruginosa* [26]. 

This paper presents a report on the coordination of Cu(II) with 1,2,4-triazole as a new ligand with potential antimicrobial activity. The method of coordinating 4-cyclohexyl-3-(4-nitrophenyl)methyl-1,2,4-triazolin-5-thione with copper(II) ions has been established. The physicochemical properties of the newly formed complex and its antimicrobial potential were tested in vitro.

## 2. Materials and Methods

### 2.1. Materials and Instruments

Chemical reagents used for the synthesis were purchased from AlfaAesar, Merck and Sigma-Aldrich. Melting points (°C) were determined using Fisher-Johns block (no correction). ^1^H, ^13^C NMR spectra were taken on a Bruker AVANCE III (300 MHz) instrument in DMSO-d_6_. Chemical shifts are reported in parts per million (ppm) relative to residual solvent peak. The mass measurements were made using an Agilent Technologies liquid chromatograph 1290, coupled to a 6550 iFunnel Q-TOF LC/MS (Agilent Technologies, Santa Clara, CA, USA). The content of Cu(II) in the solid complex was determined by the F-AAS spectrometer with a continuum source of light and using air/acetylene flame (Analityk Jena, contraAA 300). Absorbance was measured at an analytical spectral line of 324.7 nm. The limit of quantification was 0.04 mg/L. A solid sample was decomposed using the Anton Paar Multiwave 3000 closed system instrument. Mineralization was carried out for 45 min at 240 °C under pressure 60 bar. The contents of carbon, hydrogen and nitrogen were determined by a Vario micro company Elementar Analysensysteme GmbH. FTIR spectra were recorded with an IRTracer-100 Schimadzu Spectrometer (4000–600 cm^−1^) with an accuracy of recording 1 cm^−1^, using KBr pellets. The thermolysis of compounds in air atmosphere was studied by TG-DTG techniques in the range of temperature 25 to 1000 °C at a heating rate of 10 °C·min^−1^; TG and DTG curves were recorded on Netzsch TG 209 apparatus under air atmosphere v = 20 mL·min^−1^ using ceramic crucibles. As a reference material, ceramic crucibles were used. The newly synthesized compounds were studied in vitro for antimicrobial effects in accordance with the guidelines of the European Committee on Antimicrobial Susceptibility Testing (EUCAST) [29] and Clinical and Laboratory Standards Institute (CLSI) [30]. In this research, the broth microdilution method prepared in 96-well plates and reference strains of bacteria or fungi from American Type Culture Collection (ATCC) were used. These microorganisms included Gram-positive bacteria (cocci: *Staphylococcus aureus* ATCC 6538, *Staphylococcus aureus* ATCC 25923, *Staphylococcus epidermidis* ATCC 12228, *Micrococcus luteus* ATCC 10240, *Enterococcus faecalis* ATCC 29212 and bacilli: *Bacillus subtilis* ATCC 6633, *Bacillus cereus* ATCC 10876), Gram-negative bacteria (*Escherichia coli* ATCC 25922, *Proteus mirabilis* ATCC 12453, *Klebsiella pneumoniae* ATCC 13883, *Salmonella Typhimurium* ATCC 14028, *Pseudomonas aeruginosa* ATCC 9027, *Bordetella bronchiseptica* ATCC 4617) and fungi belonging to yeasts (*Candida albicans* ATCC 2091, *Candida albicans* ATCC 10231, *Candida parapsilosis* ATCC 22019, *Candida glabrata* ATCC 90030, *Candida krusei* ATCC 14243). These assays were performed as described in other data [31,32,33]. All of the microbial cultures were sub-cultured on selected nutrient agar, and bacterial and fungal suspensions were prepared in sterile 0.85% NaCl. In turn, samples with studied compounds in dimethyl sulfoxide (DMSO) were dissolved. Ciprofloxacin, vancomycin or nystatin (Sigma Aldrich, Saint Louis, MO, USA) were used as reference antimicrobial agents. The MIC (Minimal Inhibitory Concentration) of the new compounds was examined using their two-fold dilutions (from 0.48 to 1000 µg/mL) in a suitable broth medium. The bacterial or fungal suspension was added to each well with broth and the examined compounds, followed by incubation. The value of MIC was assessed spectrophotometrically. Subsequently, the MBC (Minimal Bactericidal Concentration) or MFC (Minimal Fungicidal Concentration), defined as the lowest concentration of a compound required to kill the microorganism, was studied. All the experiments were repeated and representative data are presented [31,32]. In this study, MIC > 1000 µg/mL was defined as no bioactivity, MIC = 501–1000 µg/mL as mild bioactivity, and MIC = 126–500 µg/mL as moderate bioactivity. Moreover, MBC/MIC or MFC/MIC ratios were used to determine the bactericidal/fungicidal (MBC/MIC ≤ 4, MFC/MIC ≤ 4) or the bacteriostatic/fungistatic (MBC/MIC > 4, MFC/MIC > 4) effect of the new compounds [33].

### 2.2. Preparations and Analysis

#### 2.2.1. Preparation of 4-Nitrophenylacetic Acid Hydrazide (**1**)

The ethyl 4-nitrophenylacetate (0.01 mol) was dissolved in anhydrous ethanol (10 mL) and 0.01 mol of 100% hydrazine hydrate was added. The resulting mixture was refluxed for two hours. The solution was poured into a beaker to cool. The resulting precipitate was filtered off and dried [34].

#### 2.2.2. Preparation of 4-Cyclohexyl-1-(4-nitrophenyl)acetylothiosemicarbazide (**2**)

The 4-nitrophenylacetic acid hydrazide (0.01 mol) was dissolved in 15 mL anhydrous acetonitrile and cyclohexyl isothiocyanate (0.01 mol) was added. The mixture was kept at room temperature for 5 days. After this time, a precipitate formed, which was filtered off, washed with acetonitrile and dried.

C_15_H_20_N_4_O_3_S (336.41 g/mol), yield 88%, m.p. 156–158 °C. ^1^H NMR (300 MHz, DMSO-d_6_): δ = 1.04–1.77 (m, 10H, 5CH_2_), 3.62 (s, 2H, CH_2_), 4.07 (s, 1H, CH), 7.44–8.18 (m, 4H, CH_arom_), 9.15; 9.26; 10.02 (3s, 3H, 3NH) ppm. ^13^C NMR (75.5 MHz, DMSO-d_6_): δ = 24, 25, 32, 57, 113, 122, 123, 124, 130, 140, 147, 148, 156, 167, 180 (C=S) ppm. LC-QTOF MS (*m/z*): Calculated monoisotopic mass: 336.1256, measured monoisotopic mass: 336.1260 (Supplementary Materials).

#### 2.2.3. Preparation of 4-Cyclohexyl-3-(4-nitrophenyl)methyl-1,2,4-triazolin-5-thione (**3**)

In a round-bottomed flask, 3.36 g (0.01 mol) of (**2**) was dissolved in 20 mL of 2% sodium hydroxide. The resulting mixture was heated to reflux for two hours and then cooled. The precipitated product was obtained by neutralizing the solution with a few drops of 3M hydrochloric acid. The precipitate was then filtered, dried and crystallized from methanol.

C_15_H_18_N_4_O_2_S (318.39 g/mol), yield (61%), m.p. 200–202 °C. ^1^H NMR (300 MHz, DMSO-d_6_): δ = 1.24–2.03 (m, 10H, 5CH_2_), 3.60 (s, 2H, CH_2_), 4.57 (s, 1H, CH), 6.45-8.91 (m, 4H, CH_arom_), 14.13 (s, 1H, NH) ppm. ^13^C NMR (75.5 MHz, DMSO-d_6_): δ = 25, 26, 39, 57, 113, 122, 133, 143, 156, 167, 180 (C=S) ppm. LC-QTOF MS (*m/z*): Calculated monoisotopic mass: 318.1150, measured monoisotopic mass: 318.1155 (Supplementary Materials). FTIR spectra (KBr, cm^−1^): ν(NH) 3239, 3130; ν(CH) 2960–2915; ν(CC) 1685; ν(CN) 1608, 1535; ν(NO_2_) 1405, 1350; β(CH) 1257, 1220, 1193; ν(NN) 1014; ν(CS) 970–850; γ(CH) 854, 811, 750, 705. 

#### 2.2.4. Preparation of Copper(II) 4-Cyclohexyl-3-(4-nitrophenyl)methyl-1,2,4-triazolin-5-thione complex (**Cu-3**)

Dissolving the amount of organic ligand assumed for synthesis of the copper complex required the use a reflux condenser and a magnetic stirrer for about four hours. A coordination compound with the formula [CuCl_2_(H_2_O)_2_L_2_] was obtained by mixing 0.25 mmol of organic ligand in MeOH/EtOH (*v/v* = 1/1), with 0.25 mmol of copper(II) chloride dihydrate in MeOH/EtOH (*v/v* = 1/1). The total volume of the reaction mixture was 30 mL. In order to obtain the homogenous product, the reaction mixture was stirred for eight hours. Next, the solvent was very slowly evaporated. All steps were performed at a controlled pH (6–7) and room temperature. The obtained complex was washed with 40% EtOH and then with a mixture EtOH and Et_2_O (*v/v* = 1/1). The product was air-dried at room temperature. 

C_30_H_40_N_8_S_2_O_6_CuCl_2_ (807.28 g/mol), yield (71%), anal. calculated: Cu, 7.87; C, 44.63; H, 4.99; N, 13.88. Found: Cu, 7.79; C, 44.36; H, 4.91; N, 13.58. FTIR spectra (KBr, cm^−1^): ν(OH) 3360; ν(NH) 3165, 3112, 3085; ν(CH) 2933, 2865; ν(CC) 1729, 1689; ν(CN) 1606, 1587, 1535, 1517; ν(NO_2_) 1411, 1345; β(CH) 1259, 1222, 1193, 1178; ν(NN) 1018; ν(CS) 927, 896sh; γ(CH) 854, 815, 750, 705. 

## 3. Results and Discussion

### 3.1. Synthesis

**Scheme 1:**
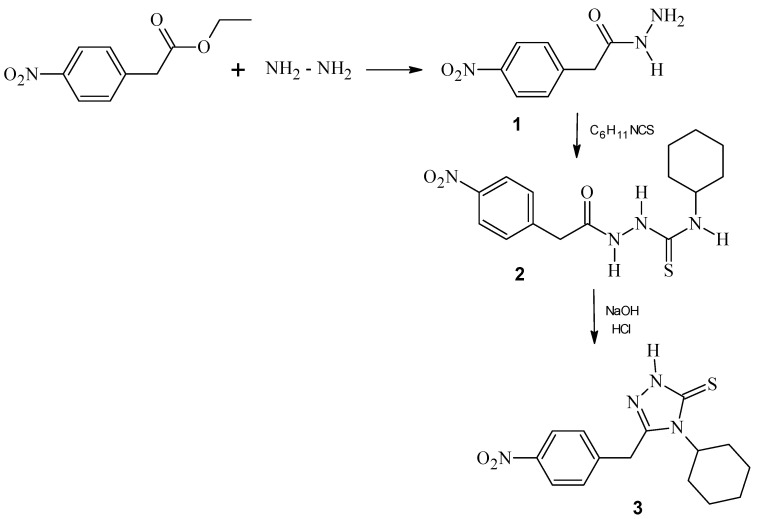
Synthesis route of the 4-cyclohexyl-3-(4-nitrophenyl)methyl-1,2,4-triazolin-5-thione (**3**).

The starting compound for the planned synthesis was 4-cyclohexyl-3-(4-nitrophenyl)methyl-1,2,4-triazolin-5-thione, which was obtained by heterocyclization of the corresponding thiosemicarbazide derivative in an alkaline medium according to Scheme 1. The newly obtained ligand was characterized by ^1^H and ^13^C NMR, FTIR spectra and TG-DTG study.

**Scheme 2:**
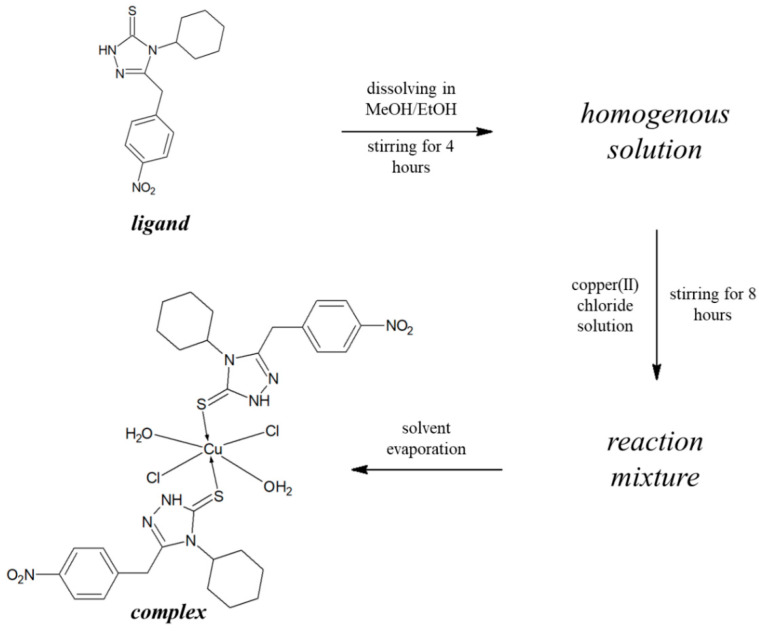
Synthesis of the copper(II) complex (**Cu-3**).

Next, the newly obtained ligand was converted to copper(II) complex. The coordination compound was obtained by the reaction of organic ligand with copper(II) chloride dihydrate in the MeOH/EtOH mixture. The synthesis is shown in Scheme 2. The newly obtained complex was characterized by elemental C/H/N analysis and Cu(II) content determination, FTIR spectra and TG-DTG study.

### 3.2. FTIR Spectra

**Figure 1:**
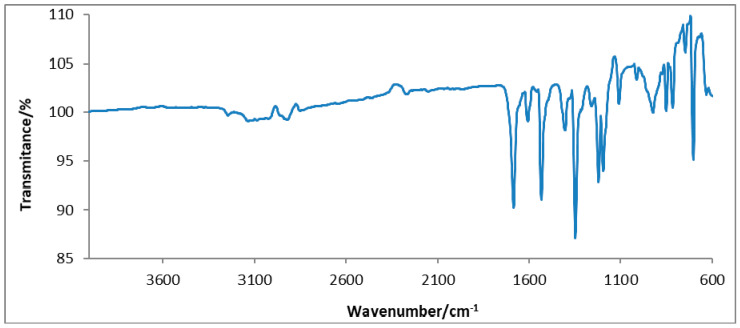
FTIR spectrum of organic ligand.

**Figure 2:**
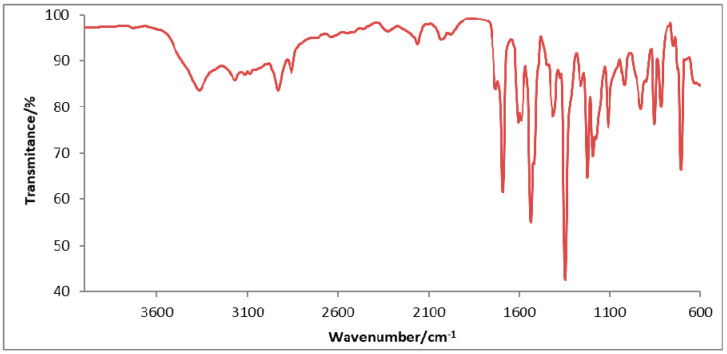
FTIR spectrum of copper(II) complex.

Figure 1 and Figure 2 present the FTIR spectrum of organic ligand and copper(II) complex, respectively. 

The spectrum of the ligand shows weak bands at 3239 and 3130 cm^−1^, due to *ν*(NH). The presence of bands at 3165, 3112 and 3085 cm^−1,^ due to *ν*(NH) in the spectrum of complex, indicates the presence of the NH group, which does not take part in bonding. A broad band form overlapping several bands observed in the range of 970 to 850 cm^−1^ ascribed to *ν*(C=S) in the free ligand appeared in the spectra of the complex as a sharp band around 927 cm^−1,^ with a shoulder at 896 cm^−1^ due to *ν*(C–S), indicating the coordination of the sulphur atom. The shifts to lower wavenumbers of *ν*(C=S) prove the copper(II)−sulphur bond. The ligand adopts thione form in the complex, which is supported by the presence of *ν*(NH) at 3165, 3112 and 3085 cm^−1^ [35,36]. The ligand shows bands at 1685 cm^−1^, due to *ν*(C=C), 1608 cm^−1^, and 1535 cm^−1^ due to *ν*(C=N), and at 1014 cm^−1^ due to *ν*(N–N). 

In the complex, the bands of ν(C=C) are observed at 1729 and 1689 cm^−1^, modes of ν(C=N) are in the range 1606−1517 and ν(N–N) at 1018 cm^−1^. The ν(NO_2_) vibrations are found at 1405 and 1350 cm^−1^ for the ligand and at 1411 and 1345 cm^−1^ for the copper compound. The CH stretching vibrations are observed between 2960−2915 cm^−1^ in the spectra of the ligand and at 2933 to 2856 cm^−1^ in the complex. For uncoordinated ligand, there are also modes of β(CH) and γ(CH) in the ranges 1257–1193 cm^−1^ and 854−705 cm^−1^, respectively. In the complex they appear in similar ranges. The occurrence of OH stretching band in the spectrum of complex at 3360 cm^−1^ indicates the presence of water molecules. Thus, it is clear from the FTIR data that the ligand acts as a monodentate ligand in the complex. 

### 3.3. Thermogravimetric Studies in Air

One of the simplest ways to determine if an obtained product is a coordination compound is to compare the thermal decomposition of the free ligand with the thermolysis of the complex. 

**Figure 3:**
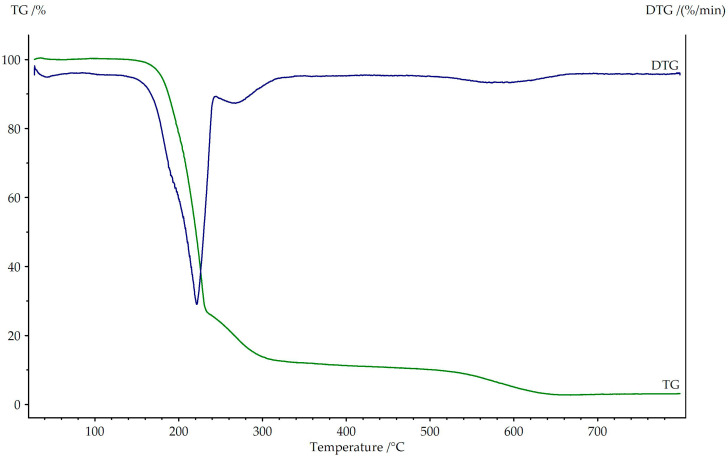
TG-DTG curves of organic ligand.

**Figure 4:**
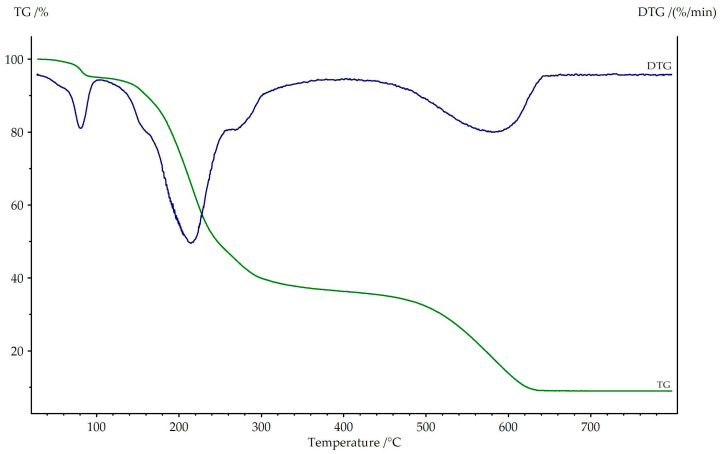
TG-DTG curves of copper(II) complex.

Both the organic ligand and the copper(II) coordination compound are stable at room temperature. Their thermal decompositions have been studied in air using TG-DTG (Figure 3 and Figure 4). Thermolysis of the ligand starts at 160 °C. In the range of 160 to 320 °C, there is a destruction of uncoordinated molecules. It is accompanied by large and small peaks on the DTG curve at 220 and 270 °C, respectively. Further temperature elevation indicates the combustion of organic residue. 

After coordination, thermal stability decreases to 50 °C. At this temperature, both water molecules are released. On the TG curve is observed a loss in mass: found. 5.00%; calc. 4.46% (DTG peak at 80 °C). Above 110 °C, decomposition of the S-donor ligand is started (mass loss: found. 54.5%; calc. 54.33%) with large peaks on the DTG curve at 210 °C. Only the fragments related to the inner coordination sphere do not take part in decomposition. In the temperature range of 310 to 640 °C, there is total destruction of the coordination compound. The DTG curve shows the presence of one peak at 580 °C. The process stops at 645 °C, with pure CuO as a final solid product of pyrolysis. Theoretical results are in very good agreement with the experimental ones (CuO found 9.5%, calculated 9.85%). The thermolysis of the complex is illustrated in Table 1. 

### 3.4. Biological Investigation

The newly synthesized ligand and its copper(II) complex were tested in vitro for antibacterial (against different reference bacteria species) and antifungal (towards yeasts from *Candida* species) activity using a broth microdilution method. The obtained results showed that these compounds exhibit some antimicrobial activity. 

The obtained data presented in Table 2 indicate that, among both new compounds, the copper(II) complex (**Cu-3**) showed higher antibacterial effects compared to the organic ligand (**3**). Gram-positive bacteria were more susceptible to the coordination compound than Gram-negative rod-shaped bacteria. The copper(II) complex (**Cu-3**) exhibited moderate activity with minimal inhibitory concentrations (MICs) ranging from 250 µg/mL to 500 µg/mL and minimal bactericidal concentrations (MBCs) from 250 µg/mL to >1000 µg/mL against reference staphylococci, micrococci and bacilli. Taking into account the MBC/MIC = 1–>2 ratios, mainly its bactericidal effect was shown. In the case of organic ligand (**3**), MIC and MBC values were lower and comparable (MIC or MBC ≥ 1000 µg/mL, MBC/MIC ≥ 1) and its activity was mild.

Coordination compound (**Cu-3**) and organic ligand (**3**) also had some activity towards reference Gram-negative microorganisms. Among them, *Bordetella bronchiseptica* ATCC 4617 was the most sensitive (MIC and MBC were 500 µg/mL and 1000 µg/mL, respectively) on tested substances. These compounds showed the same moderate activity and bactericidal effect (MBC/MIC = 2) towards this rod-shaped bacteria. The antimicrobial activity of both newly synthesized compounds against the remaining Gram-negative bacteria was lower (MIC or MBC ≥ 1000 µg/mL). Generally, these microorganisms were more resistant to the studied copper(II) complex (**Cu-3**) and organic ligand (**3**) than the Gram-positive bacteria. The antimicrobial activity of both compounds against these bacteria was mild. This may be related to the construction of their cell wall, which is much more complex, making these bacteria less sensitive to biologically active agents. 

Yeasts, belonging to the *Candida* species, especially *Candida albicans* ATCC 10231 also showed similar sensitivity, both to the copper(II) complex (**Cu-3**) and the organic ligand (**3**). The anticandidal activity of these compounds was moderate. Their minimal concentrations, which inhibited growth (MIC) or killed these fungi (MFC), were 500 µg/mL and 1000 µg/mL, respectively. Moreover, the ratio MFC/MIC = 2 indicated a fungicidal effect towards this strain. In the case of other *Candida* spp., antifungal activity was lower (MIC or MFC ≥ 1000 µg/mL). 

Noteworthy is the fact that bacteria or yeasts used in these studies are opportunistic or pathogenic microflora for the human body. Therefore, it seems practical to use the copper(II) complex (**Cu-3**) in the prevention and treatment of infections caused by these selected microorganisms.

## 4. Conclusions

New organic ligand (**3**) was obtained in the cyclization reaction of 4-cyclohexyl-1-(4-nitrophenyl)acetylothiosemicarbazide (**2**) in alkaline medium. The coordination compound (**Cu-3**) was received in the reaction of the ligand with copper(II) chloride dihydrate. It is stable at room temperature. Changes observed in the FTIR spectra of the complex indicate that 4-cyclohexyl-3-(4-nitrophenyl)methyl-1,2,4-triazolin-5-thione coordinates to metal (II) ion as a monodentate ligand via the S-donor atom. The shape of the TG curve, solid intermediate and final products, clearly indicate the obtainment of the coordination compound. The test in vitro for antibacterial and antifungal activity indicated that the copper(II) complex (**Cu-3**) had some antimicrobial activity with moderate effect, especially against Gram-positive bacteria. Additionally, this complex showed higher antibacterial effect compared to the organic ligand (**3**). It is very likely that further modifications will improve the performances of both the free ligand and the obtained complex, especially as an antibacterial agent. In this context, both 1,2,4-triazole derivatives and copper can be used as antimicrobial agents, and at the same time, be used to synthesize new antimicrobial drugs, such as complexes copper(II) with 1,2,4-triazole derivatives (as a new ligand). In turn, the newly obtained compounds may be regarded as precursor agents in the search for other derivatives with better antimicrobial activity.

## Figures and Tables

**Table 1:** TG-DTG analysis data for decomposition process of copper(II) complex.

Compound	Range of Decomposition (°C)	DTG Peak (°C)	Mass Loss (%)		Intermediate Product or Solid Residue
			Found	Calc.	
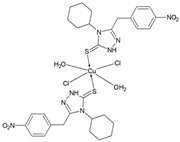	50–110	80	5.0	4.46	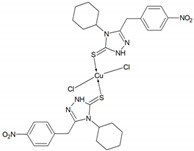
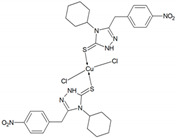	110–310	210	54.5	54.33	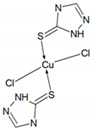
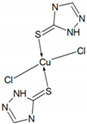	310–640	580	31.0	31.36	CuO

**Table 2:** The activity data of both compounds: organic ligand (**3**) and copper(II) complex (**Cu-3**) towards some of the reference microorganisms.

Species	MIC (MBC or MFC) [µg/mL] and {MBC/MIC or MFC/MIC} of Compounds		
	Copper(II) Complex (Cu-3)	Organic Ligand (3)	CIP/NY *
*Staphylococcus aureus* ATCC 25923	250 (250) {1}	1000 (>1000) {>1}	0.48 (0.48) {1}
*Staphylococcus aureus* ATCC 29213	250 (250) {1}	>1000 (>1000) {>1}	0.48 (0.48) {1}
*Stphylococcus epidermidis* ATCC 12228	250 (250) {1}	1000 (1000) {1}	0.12 (0.12) {1}
*Micrococcus luteus* ATCC 10240	250 (250) {1}	1000 (1000) {1}	0.98 (1.95) {2}
*Bacillus cereus* ATCC 10876	500 (>1000) {>2}	1000 (>1000) {>1}	0.06 (0.12) {2}
*Bacillus subtilis* ATCC 6633	250 (250) {1}	1000 (>1000) {>1}	0.03 (0.03) {1}
*Bordetella bronchiseptica* ATCC 4617	500 (1000) {2}	500 (1000) {2}	0.98 (0.98) {1}
*Candida albicans* ATCC 10231	500 (1000) {2}	500 (1000) {2}	0.48 * (0.48) {1}

The standard antibiotics were used as positive controls: Ciprofloxacin (CIP) against bacteria and nystatin (NY *) against yeasts.

## Supplementary Materials

The following are available online at https://www.mdpi.com/1996-1944/13/18/4135/s1, Figure S1: ^1^H NMR spectrum for compound 2, Figure S2: ^13^C NMR spectrum for compound 2, Figure S3: ^1^H NMR spectrum for compound 3, Figure S4: ^13^C NMR spectrum for compound 3, Figure S5: (a) The structure of compound 2; (b) The structure of compound 3; (c) MS spectrogram for compound 2; (d) MS spectrogram for compound 3.
