# Supplementary material for: Synthesis, Spectral, Thermal and Biological Studies of 4-Cyclohexyl-3-(4-nitrophenyl)methyl-1,2,4-triazolin-5-thione and Its Copper(II) Coordination Compound, [CuCl2(H2O)2L2]
Source: Materials (Basel). 2020 Sep 17;13(18):4135. doi: 10.3390/ma13184135 (PMC7560296; doi:10.3390/ma13184135)
Supplement: Supplementary file 1 [file materials-13-04135-s001.pdf]

Synthesis, Spectral, Thermal and Biological Studies of 4-Cyclohexyl-3-(4-nitrophenyl)methyl-1,2,4-triazolin-5-thione and Its Copper(II) Coordination Compound, [$CuCl_2(H_2O)_2L_2$]

Agnieszka Czylkowska [1,*], Monika Drozd [2], Anna Biernasiuk [3], Bartłomiej Rogalewicz [1], Anna Malm [3] and Monika Pitucha [2]

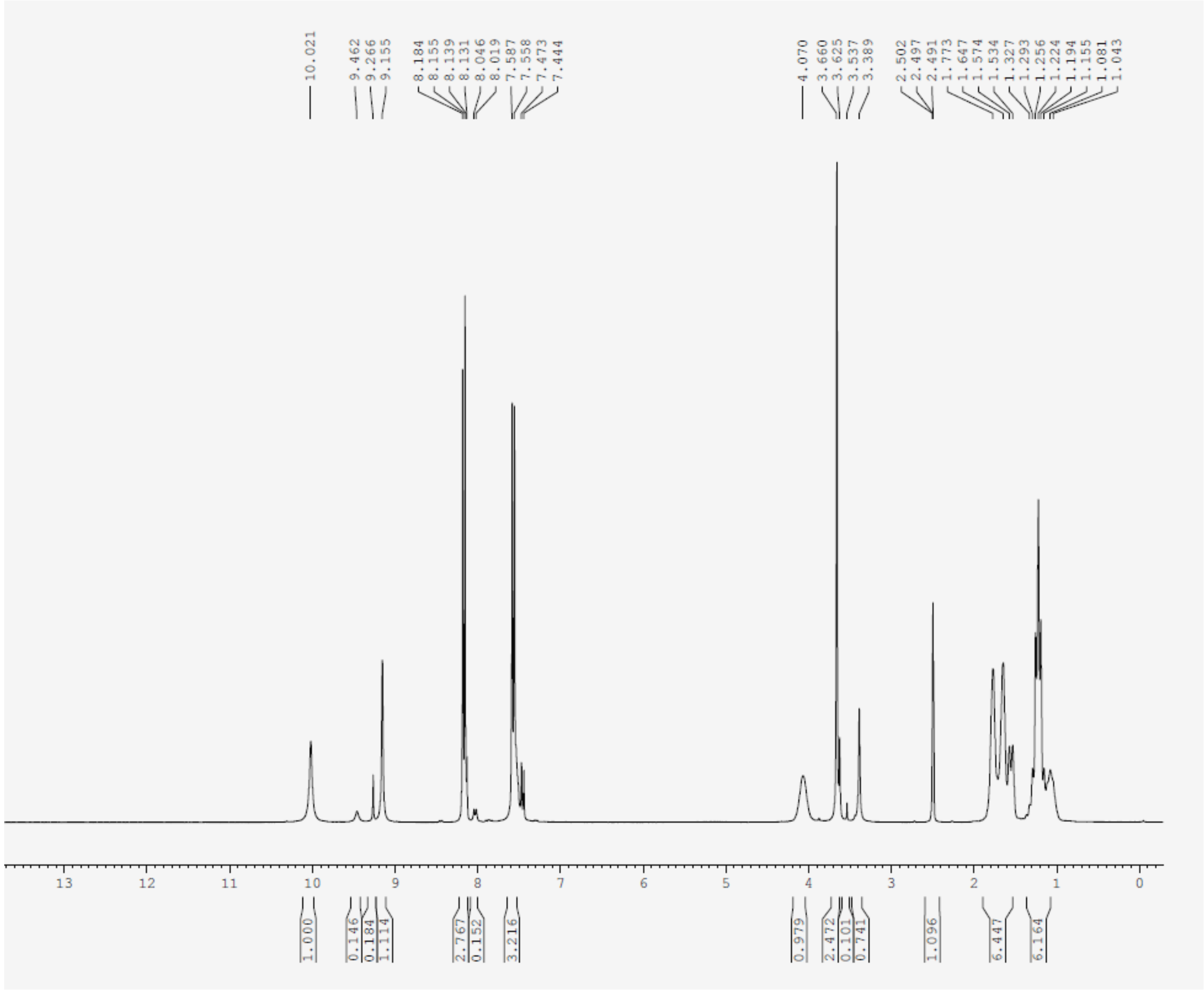

Figure S1. ^{1}H NMR spectrum for compound **2**.

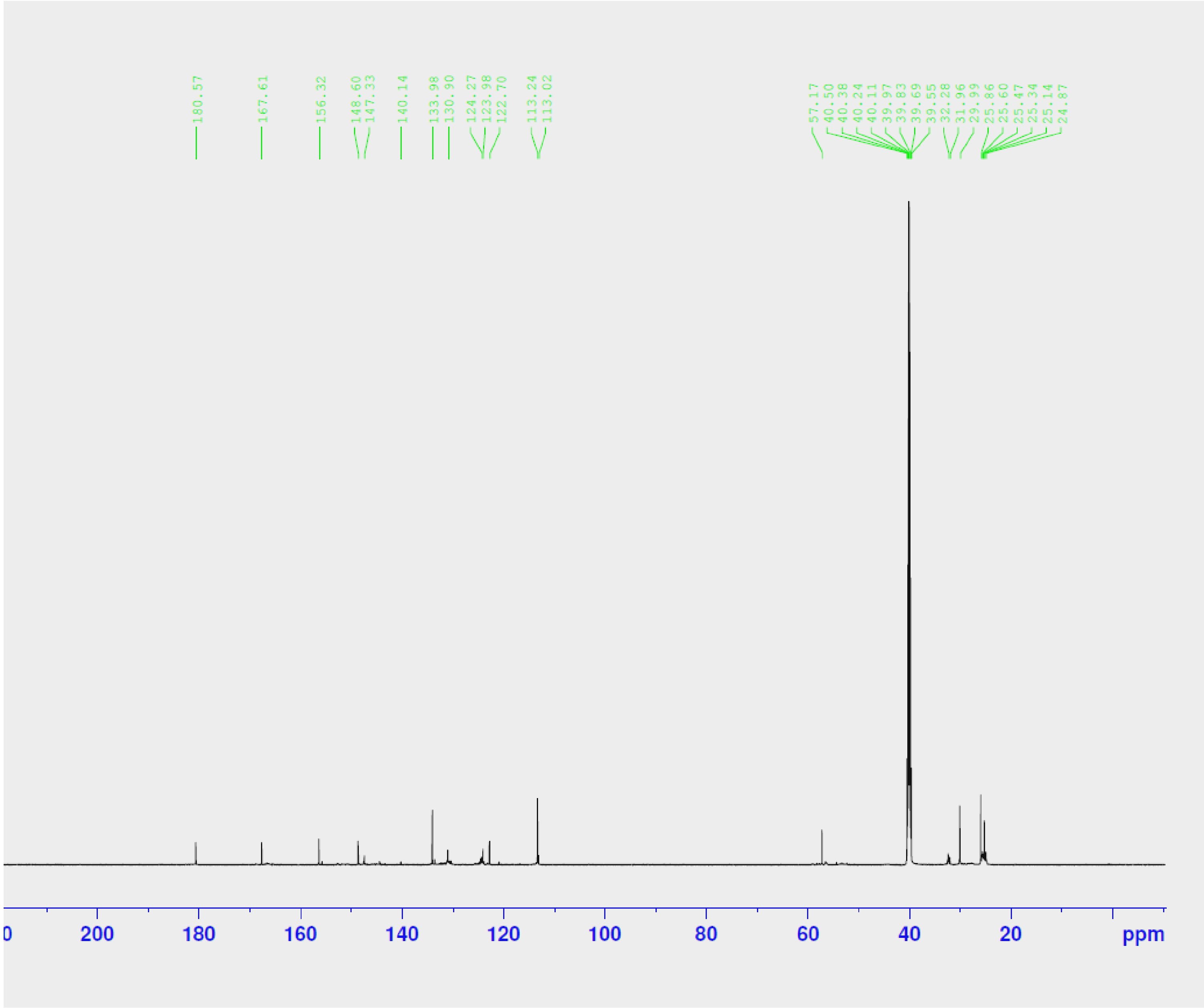

Figure S2. ^{13}C NMR spectrum for compound **2**.

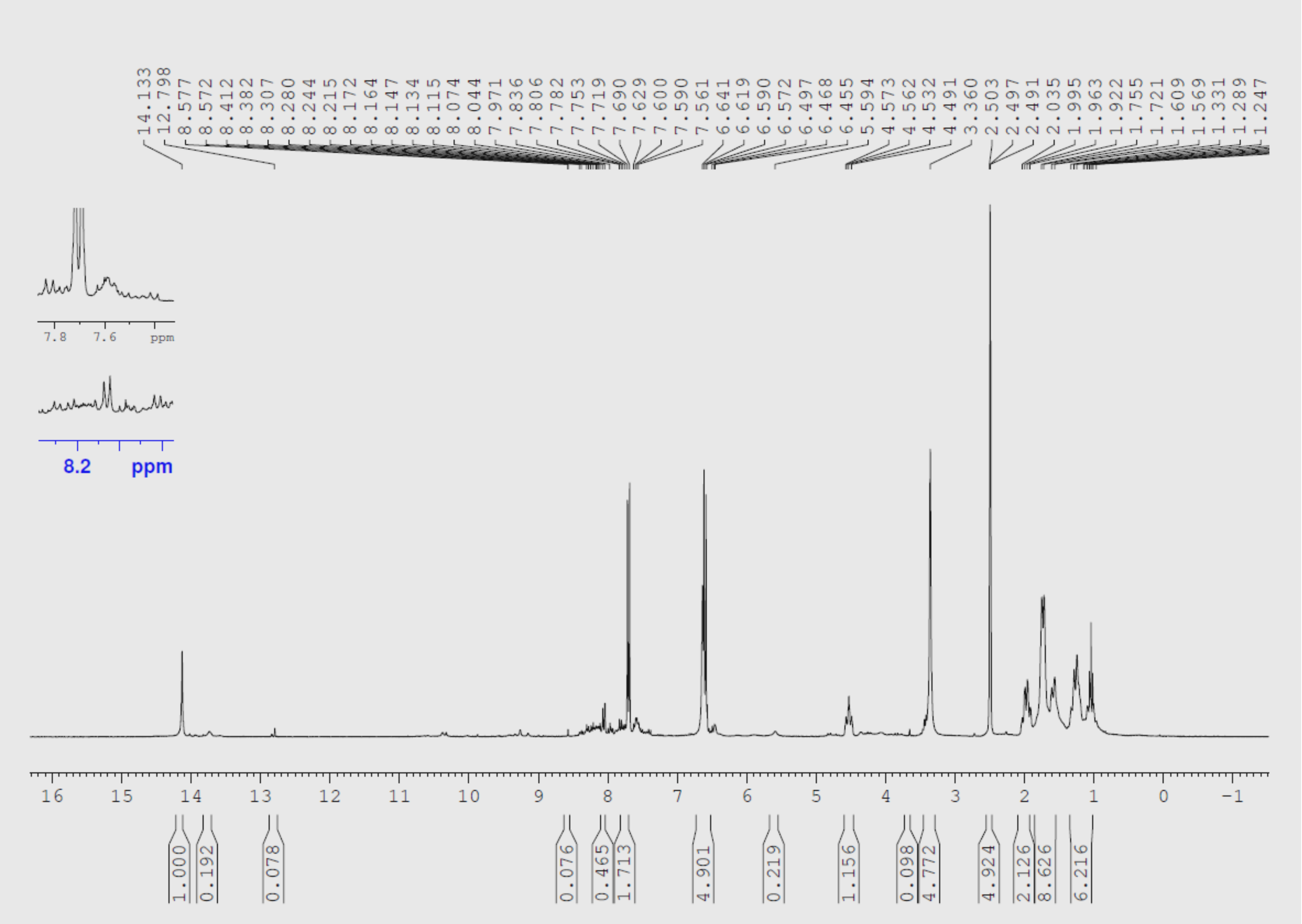

Figure S3: ^{1}H NMR spectrum for compound **3**.

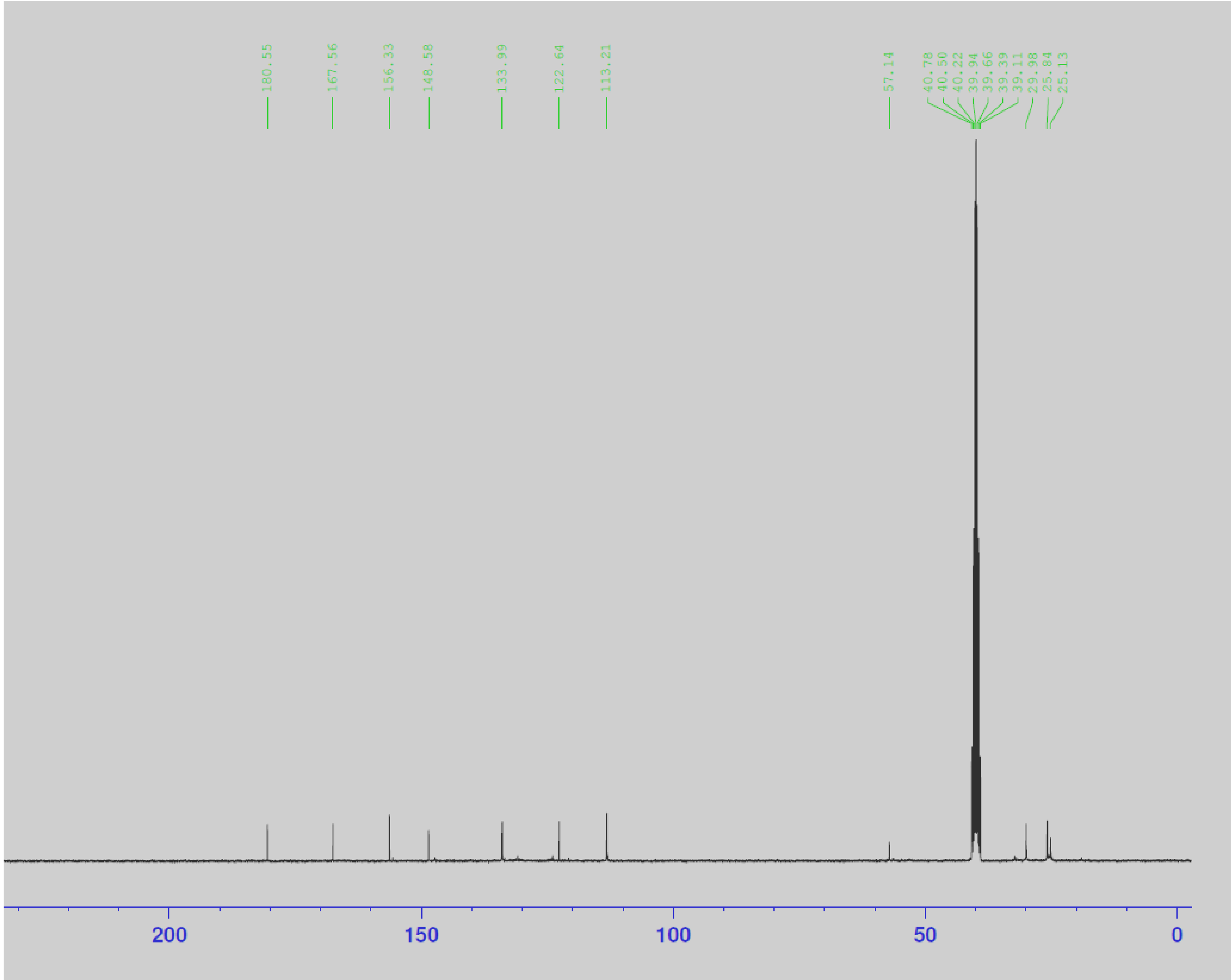

Figure S4: ^{13}C NMR spectrum for compound **3**.

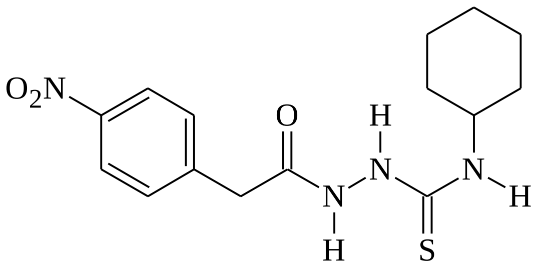

$C_{15}H_{20}N_4O_3S$

Calculated monoisotopic mass: 336.1256
Measured monoisotopic mass: 336.1260
Mass error: 1.24 ppm

(**a**)

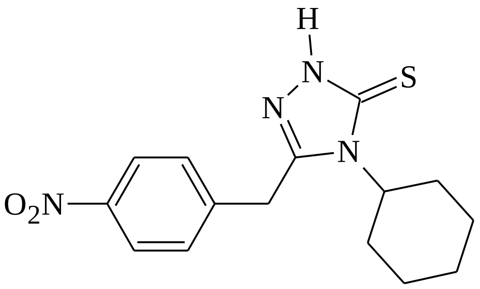

$C_{15}H_{18}N_4O_2S$

Calculated monoisotopic mass: 318.1150
Measured monoisotopic mass: 318.1155
Mass error: 1.36 ppm

(**b**)

Theoretical monoisotopic mass of $[M+H]^+$: 337.1329

LC/QTOF MS scan

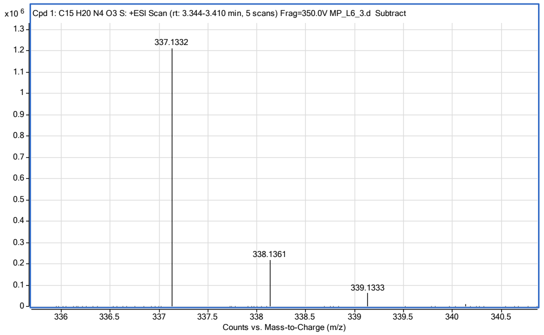

(**c**)

Theoretical monoisotopic mass of $[M+H]^+$: 319.1223

LC/QTOF MS scan

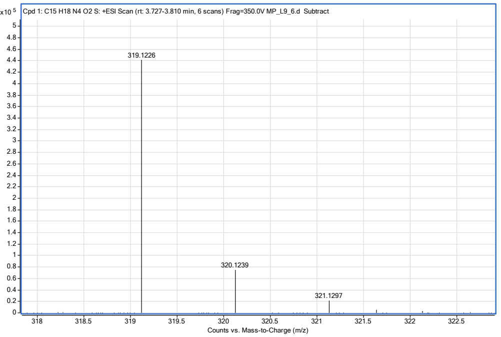

(**d**)

Figure S5. (**a**) The structure of compound **2;** (**b**) The structure of compound 3; (**c**) MS spectrogram for compound **2**; (**d**) MS spectrogram for compound **3.**

© 2020 by the authors. Submitted for possible open access publication under the terms and conditions of the Creative Commons Attribution (CC BY) license (http://creativecommons.org/licenses/by/4.0/).
